# Responding to the Pandemic: An Update From the GSA COVID-19 Task Force

**DOI:** 10.1093/geroni/igaa057.2942

**Published:** 2020-12-16

**Authors:** Kathryn Hyer, Elizabeth Sobczyk

## Abstract

The GSA COVID-19 Task Force was convened in March 2020 to address the needs of GSA members and older adults related to the COVID-19 pandemic. This interdisciplinary group of members represents physicians, nurses, social workers, psychologists, educators, and researchers with expertise in disaster preparedness, caregiving, trauma, HIV, vaccination, home health, long-term care, social isolation, motivation, and risk communication with older adults. In this symposium, Task Force members will share how they have advanced the areas of research, education, and practice related to the pandemic. Speakers will discuss the process of developing a COVID-19 research agenda and how that is moving forward. They will also share how Task Force members representing AGHE have been supporting members through listening sessions and the development of materials to assist with the transition to online teaching. Finally, speakers will provide updates on the several fact sheets, webinars, and a decision aid that have been developed to support GSA members and their work with older adults.

